# The vasoprotective role of IGF-1 signaling in the cerebral microcirculation: prevention of cerebral microhemorrhages in aging

**DOI:** 10.1007/s11357-024-01343-5

**Published:** 2024-09-14

**Authors:** Levente Stankovics, Anna Ungvari, Mónika Fekete, Adam Nyul-Toth, Peter Mukli, Roland Patai, Boglarka Csik, Rafal Gulej, Shannon Conley, Anna Csiszar, Peter Toth

**Affiliations:** 1 Department of Neurosurgery, Medical School, University of Pecs, Pecs, Hungary; 2 Institute of Preventive Medicine and Public Health, Semmelweis University, Budapest, Hungary; 3 International Training Program in Geroscience, Doctoral College-Health Sciences Program/ Institute of Preventive Medicine and Public Health, Semmelweis University, Budapest, Hungary; 4 Vascular Cognitive Impairment, Neurodegeneration and Healthy Brain Aging Program, Department of Neurosurgery, University of Oklahoma Health Sciences Center, Oklahoma City, OK USA; 5 Department of Cell Biology, University of Oklahoma Health Sciences Center, Oklahoma City, OK USA

**Keywords:** Microbleed, Cerebral small vessel disease, CSVD, Insulin-like Growth Factor 1, Aging, Hypertension, Hypertensive

## Abstract

Aging is closely associated with various cerebrovascular pathologies that significantly impact brain function, with cerebral small vessel disease (CSVD) being a major contributor to cognitive decline in the elderly. Consequences of CSVD include cerebral microhemorrhages (CMH), which are small intracerebral bleeds resulting from the rupture of microvessels. CMHs are prevalent in aging populations, affecting approximately 50% of individuals over 80, and are linked to increased risks of vascular cognitive impairment and dementia (VCID). Hypertension is a primary risk factor for CMHs. Vascular smooth muscle cells (VSMCs) adapt to hypertension by undergoing hypertrophy and producing extracellular matrix (ECM) components, which reinforce vessel walls. Myogenic autoregulation, which involves pressure-induced constriction, helps prevent excessive pressure from damaging the vulnerable microvasculature. However, aging impairs these adaptive mechanisms, weakening vessel walls and increasing susceptibility to damage. Insulin-like Growth Factor 1 (IGF-1) is crucial for vascular health, promoting VSMC hypertrophy, ECM production, and maintaining normal myogenic protection. IGF-1 also prevents microvascular senescence, reduces reactive oxygen species (ROS) production, and regulates matrix metalloproteinase (MMP) activity, which is vital for ECM remodeling and stabilization. IGF-1 deficiency, common in aging, compromises these protective mechanisms, increasing the risk of CMHs. This review explores the vasoprotective role of IGF-1 signaling in the cerebral microcirculation and its implications for preventing hypertension-induced CMHs in aging. Understanding and addressing the decline in IGF-1 signaling with age are crucial for maintaining cerebrovascular health and preventing hypertension-related vascular injuries in the aging population.

## Introduction

Aging is associated with various cerebrovascular pathologies that significantly impact higher brain functions [1, 2]. Among these challenges, cerebral small vessel disease (CSVD) is a major contributor to the decline in brain health observed in the elderly [3, 4]. CSVD encompasses a range of pathologies affecting the small blood vessels in the brain, including arterioles, capillaries, and venules [5]. A critical manifestation of CSVD is the development of cerebral microhemorrhages (CMH), which are small intracerebral bleeds that occur due to the rupture of cerebral microvessels [3, 6–11].

CMHs are typically identified using MRI techniques such as T2*-weighted gradient-recall echo (GRE) or susceptibility-weighted imaging (SWI), where they appear as small, round, hypointense regions [6, 9, 12]. These imaging signs of CMHs are a hallmark of CSVD and are prevalent in aging populations [12]. Studies have shown that the prevalence of CMHs increases with age, affecting up to 23% of individuals over the age of 60 and over 50% of those over the age of 80 [12]. The presence of multiple CMHs is often associated with a higher risk of cognitive decline, gait dysfunction, and the development of vascular cognitive impairment and dementia (VCID), thereby significantly reducing the quality of life in elderly individuals [8, 12–14].

CMHs are a part of the broader spectrum of pathological consequences of CSVD, which also include white matter hyperintensities, lacunar infarcts, and enlarged perivascular spaces [3, 12]. Together, these conditions contribute to a progressive deterioration of cognitive function, leading to increased disability and dependency in the elderly [12]. The pathophysiology of CMHs involves the weakening of the vascular wall, often exacerbated by chronic hypertension [2, 15] and other cardiovascular risk factors [3, 12]. This weakening results in increased fragility of the microvasculature, making it susceptible to rupture under stress [12]. The pathomechanisms contributing to the genesis of CMHs are likely multifaceted [12].

Insulin-like Growth Factor 1 (IGF-1) is a critical anti-aging growth factor that plays a vital role in maintaining vascular health [16–33]. IGF-1 levels naturally decline with age, which may contribute to the increased incidence of CMH and other cerebrovascular pathologies in the elderly [30]. IGF-1 exerts vasoprotective effects by promoting the survival and preserving the physiological functions of vascular cells, including endothelial cells and vascular smooth muscle cells (VSMCs) [16, 34]. By enhancing the structural integrity and adaptive capacity of these cells, IGF-1 helps to preserve the function of the microvasculature and prevent the development of CMHs and other manifestations of CSVD [16, 34].

This mini-review discusses the vasoprotective role of IGF-1 signaling in VSMCs and its implications for preventing hypertension-induced CMHs in aging. By understanding the mechanisms through which IGF-1 contributes to vascular health, we can develop targeted therapeutic strategies to mitigate the impact of CSVD and improve the quality of life for the aging population.

## Structural and functional cerebromicrovascular adaptations to hypertension: role of aging

Hypertension induces microvascular remodeling, a process involving structural and functional changes in resistance arterioles to accommodate increased blood pressure [2, 12, 35–38]. This remodeling is crucial for maintaining microvascular integrity and preventing damage to the blood vessel walls [15, 39, 40]. Vascular smooth muscle cells (VSMCs) play a pivotal role in this process by undergoing hypertrophy and producing extracellular matrix (ECM) components that reinforce the vessel walls. Additionally, VSMCs regulate microvascular tone [33, 35, 37, 41–45], thereby increasing cerebrovascular resistance [1, 33, 37].

A key component of this adaptive response is myogenic autoregulation, which involves pressure-induced myogenic constriction [1, 33, 37, 45]. Myogenic tone refers to the intrinsic ability of blood vessels, particularly arterioles, to constrict in response to increases in intraluminal pressure. This autoregulatory response helps to stabilize blood flow and prevent excessive pressure from reaching the vulnerable, distal portions of the microvasculature, such as capillaries, which are prone to rupture under high pressure [2, 37]. Effective myogenic tone adaptation ensures that the cerebral microcirculation remains protected from the damaging effects of hypertension [2, 37]. In response to chronic hypertension, the myogenic constriction of cerebral vessels increases, shifting the autoregulatory range to higher pressures [1, 33, 37]. This homeostatic adaptation protects the vulnerable downstream portions of the microcirculation from the penetration of high-pressure waves. By increasing cerebrovascular resistance, VSMCs help to maintain a stable microenvironment for the brain's delicate tissues, thus preventing the formation of CMHs [1, 33, 37]. These structural and functional adaptations are essential to counteract the adverse effects of sustained high blood pressure and protect the microvascular networks within the brain.

Aging leads to reduced hypertension-induced VSMC hypertrophy and inadequate ECM production. Aging also up-regulates hypertension-induced ROS production [46] and activation of ECM degrading MMPs [36]. This results in weaker vessel walls that are more susceptible to damage from increased blood pressure [36]. Additionally, the aging process affects the ability of VSMCs to regulate myogenic tone [1, 35, 37]. The intrinsic myogenic response becomes less effective, and the autoregulatory range does not shift adequately to higher pressures, making the microcirculation more vulnerable to the damaging effects of hypertension [1, 37]. Preclinical studies suggest that aged hypertensive mice lack the adaptive increases in pressure-induced calcium signaling and myogenic tone seen in cerebral arteries of young hypertensive mice, which is attributed to dysregulation of 20-HETE and TRP channel-mediated signaling [35]. The cumulative impact of these age-related impairments is an increased risk of CMH [1, 37]. As the vessel walls weaken and the ability to regulate intraluminal pressure deteriorates, the microvasculature becomes prone to ruptures, leading to CMHs [1, 37]. These microbleeds can have significant consequences, including cognitive decline and motor impairments, which are commonly observed in the elderly population.

## Structural and functional cerebromicrovascular adaptations to hypertension: role of IGF-1 deficiency

The importance of IGF-1 in supporting protective microvascular adaptations to hypertension cannot be overstated [20, 23, 33]. IGF-1 plays a critical role in VSMCs, promoting adaptive hypertrophy characterized by the enlargement of VSMCs and increased production of extracellular matrix (ECM) proteins and cross-linking enzymes [20]. IGF-1 signaling enhances the expression of key contractile proteins and ECM components, such as collagen and elastin, which are necessary for maintaining vascular structure and function [20]. Additionally, IGF-1 prevents microvascular senescence, reduces reactive oxygen species (ROS) production [17, 30], and regulates the activity of matrix metalloproteinases (MMPs) [30], all of which are critical for ECM remodeling and stabilization. Moreover, IGF-1 signaling enhances the contractile function of VSMCs, enabling them to maintain proper myogenic tone and thus protect the microvasculature from excessive pressure [33]. Through these processes, IGF-1 helps maintain a resilient microvascular network capable of withstanding elevated blood pressure.

IGF-1 exerts its vasoprotective effects through the IGF-1 receptor (IGF-1R) expressed on various cell types, including VSMCs and endothelial cells. Binding of IGF-1 to IGF-1R activates multiple signaling pathways such as the PI3K/Akt and MAPK/ERK pathways, which are crucial for cell survival, proliferation, and differentiation. In VSMCs, IGF-1 promotes a protective phenotype, characterized by optimal structural remodeling in response to mechanical stress and increased expression of ECM remodeling genes. IGF-1 also exerts antioxidative and anti-senescence effects and regulates pathways involved in cellular stress resilience [17], including DNA repair pathways. Phenotypic switching in VSMCs, a process where cells transition between a contractile phenotype and a variety of synthetic or proliferative phenotypes, is essential for normal VSMC function. The contractile VSMC phenotype is essential for vascular tone and stability, while various growth factors and other stimuli can promote adoption of synthetic phenotypes associated with ECM production and repair, or maladaptive, inflammatory phenotypes that contribute to cardiovascular and cerebrovascular disease [47–53]. IGF-1 influences this phenotypic switching toward a protective and adaptive state, thereby enhancing the structural integrity and function of blood vessels under stress conditions such as hypertension [23, 54]. The role of IGF-1 in structural remodeling is fundamental, ensuring the vascular walls are adequately reinforced and functional.

Experimental studies support the central role of IGF-1 in protecting against CMHs [23]. In IGF-1 deficient mouse models [24, 29] and mice with genetic disruption of IGF-1 signaling (such as VSMC-specific knockdown of the IGF-1 receptor [23]), the absence of these adaptive mechanisms leads to increased vascular fragility and susceptibility to microhemorrhages. The absence of adequate IGF-1 signaling leads to reduced VSMC hypertrophy and ECM production, resulting in weaker vessel walls that are more susceptible to damage [23, 29]. IGF-1 deficiency negatively impacts the expression of key contractile proteins and ECM components, such as collagen and elastin, which provide the structural support needed to maintain vascular integrity under hypertensive conditions [20]. IGF-1 deficient mice exhibit increased pressure-induced vascular ROS production and activation of MMPs, exacerbating vascular damage [29]. Strong evidence suggests that IGF-1 signaling can attenuate the generation of ROS both by NADPH oxidases like Nox4 and mitochondria [17, 18, 30, 55–60]. It also enhances the expression of antioxidant enzymes and reduces the overall oxidative burden within cells, thereby mitigating the damaging effects of excessive ROS production.

Myogenic tone adaptation involves several molecular pathways that are influenced by IGF-1 signaling [33]. The lack of adaptive hypertrophy in animal models with disrupted IGF-1 signaling [20] is associated with a compromised ability of VSMCs to effectively regulate myogenic tone, allowing high blood pressure to penetrate into the microvasculature. Consequently, the distal portions of the microvasculature, including capillaries, are exposed to damaging high pressure, increasing the risk of CMHs, mimicking the aging phenotype.

In conclusion, the ability of the vascular smooth muscle to adapt to hypertension through structural and functional changes is vital for protecting the cerebral microvasculature [20, 23, 24, 29]. Myogenic autoregulation, supported by IGF-1 signaling, plays a crucial role in preventing the penetration of high-pressure waves into the brain's microcirculation, thereby preventing the genesis of CMHs. The importance of IGF-1 and specifically IGF-1 in VSMCS in preventing CMH is highlighted by preclinical studies clearly demonstrating that reduction in circulating IGF-1 levels, or VSMC knockout of Igf1 receptor accelerates and exacerbates the development of CMH [23, 24, 30]. The decline in IGF-1 levels with aging likely plays a significant role in the impaired structural and functional adaptations to hypertension. This underscores the importance of maintaining adequate IGF-1 signaling to support cerebrovascular health, particularly in the context of aging and hypertension. Understanding the age-related impairments in these adaptive mechanisms highlights the need for therapeutic strategies that enhance IGF-1 signaling to maintain cerebrovascular health and prevent the adverse effects of hypertension in the aging population.

Moreover, IGF-1 exerts protective effects on other cells of the neurovascular unit, including endothelial cells and astrocytes [21, 27, 28, 32]. IGF-1 has been shown to protect endothelial function, support endothelium and astrocyte-mediated neurovascular coupling responses, and promote capillarization and blood–brain barrier (BBB) integrity [16, 21]. IGF-1 signaling is vital for the proper functioning of the endothelium, particularly through its regulation of eNOS [61–63]. IGF-1 enhances eNOS expression and activity, leading to increased NO production. This process is crucial for maintaining the integrity of the cerebral microvasculature, as NO not only promotes vasodilation but also inhibits leukocyte adhesion and platelet aggregation, thereby reducing the risk of vascular inflammation and thrombosis. Furthermore, NO produced by eNOS is essential for the maintenance of the BBB, which maintains the homeostatic environment necessary for proper neural function. By maintaining endothelial health and enhancing the interactions between endothelial cells and astrocytes, IGF-1 helps to preserve the overall function and stability of the neurovascular unit [16, 27–29, 32]. These protective effects further underscore the importance of IGF-1 in maintaining brain health and preventing CSVD, particularly in the context of aging and hypertension.

## Prevention of cerebral microhemorrhages

Effective prevention of CMHs in aging is multifaceted, requiring both control of hypertension and maintenance of microvascular resilience [12]. This includes sustaining normal IGF-1 levels for optimal cerebromicrovascular and overall brain health.

Hypertension is a primary risk factor for the development of CMHs [12]. Elevated blood pressure exerts continuous stress on the vascular system, particularly on the delicate microvessels in the brain. Controlling hypertension is therefore crucial to prevent CMHs and associated cognitive and motor impairments. Effective management of hypertension involves lifestyle modifications such as a balanced diet, regular physical activity, and weight management. Pharmacological treatments, including antihypertensive medications like ACE inhibitors and calcium channel blockers, are also essential [12]. These interventions help to lower blood pressure, thereby reducing the mechanical stress on cerebral vessels and preventing the formation of microbleeds. In addition to these strategies, it is important to prevent sudden blood pressure surges, which can exacerbate the risk of CMH. Activities that involve straining, such as the Valsalva maneuver [64], lifting heavy objects or exerting force while on the toilet, should be approached with caution. Adopting proper techniques and avoiding excessive exertion during these activities can help prevent acute increases in blood pressure that might compromise the integrity of the cerebral microvasculature. Additionally, maintaining loose stools through adequate hydration, fiber intake, and the use of stool softeners when necessary can reduce straining during bowel movements, further helping to prevent sudden spikes in blood pressure.

Normal IGF-1 levels are essential for ensuring the structural and functional health of the brain's microvasculature. In aging populations, where IGF-1 levels naturally decline, maintaining normal IGF-1 levels can mitigate the risks associated with vascular fragility and CMH. While IGF-1 is beneficial for vascular health, there are significant concerns regarding its use as a treatment due to its association with increased cancer risk. IGF-1 is a potent growth factor that can promote cellular proliferation, and elevated levels have been linked to the growth of certain cancers. This has led to a cautious approach in recommending IGF-1 treatments, particularly for long-term use. Given the limitations of direct IGF-1 treatment, alternative strategies to maintain healthy IGF-1 levels are of great interest. Lifestyle and dietary interventions have shown promise in modulating IGF-1 levels naturally. Among them, certain dietary patterns can influence IGF-1 levels. Diets rich in protein have been associated with higher IGF-1 levels. A balanced approach that includes moderate protein intake, healthy fats, and complex carbohydrates can support overall health and potentially beneficial IGF-1 levels. Regular physical activity is a well-known modulator of IGF-1. Both aerobic and resistance training exercises can increase IGF-1 levels, promoting vascular health and supporting the maintenance of muscle mass and function [65–70]. Ensuring proper endocrine function, including the management of growth hormone levels, can also help maintain adequate IGF-1 production. Hormone replacement therapies, under medical supervision, might be an option for some individuals to support IGF-1 levels. Certain supplements, such as vitamin D [71] and omega-3 fatty acids [72], have been linked to the regulation of IGF-1. Ensuring adequate intake of these nutrients can support IGF-1 production and overall cerebrovascular health.

In conclusion, preventing CMHs requires a comprehensive approach that includes controlling hypertension and maintaining healthy IGF-1 levels. While direct IGF-1 treatment poses significant risks, lifestyle and dietary interventions offer promising alternatives for supporting IGF-1 levels and promoting cerebrovascular health. Further research into these strategies and their long-term impacts will be essential for developing effective prevention and treatment protocols for CMH and other age-related cerebrovascular pathologies.

## Future perspectives

Future research should delve deeply into elucidating the precise mechanisms through which IGF-1 signaling confers vasoprotection. Understanding these mechanisms will facilitate the development of targeted therapies aimed at enhancing IGF-1 signaling specifically in VSMCs and endothelial cells. Additionally, long-term studies are necessary to evaluate the effectiveness of these therapies in preventing not only CMHs but also other age-related cerebrovascular pathologies. By comprehending and harnessing the vasoprotective role of IGF-1, we can better address the challenges posed by cerebrovascular aging, thereby improving the health span and quality of life for the elderly population.

A critical area of study is the cellular processes influenced by IGF-1. Researchers need to examine how IGF-1 affects VSMC phenotypic switching between contractile and synthetic states, and how this switching impacts vascular remodeling and stability. Additionally, exploring IGF-1's role in maintaining BBB integrity and preventing cellular senescence that can contribute to CMHs [73] will be essential. Understanding the interactions between paracrine IGF-1 and cells of the neurovascular unit, such as endothelial cells, pericytes, and astrocytes, will further elucidate the comprehensive vasoprotective effects of IGF-1.

Emerging evidence suggests that IGF-1 signaling in the cerebral microcirculation exhibits sex-dependent differences, particularly in elderly subjects. Sex differences in IGF-1 levels are well-documented, with males generally having higher circulating IGF-1 levels than women. Both sexes experience an age-related decline in IGF-1, but this decline is often more pronounced in women following menopause due to the significant drop in estrogen. Estrogen has been shown to enhance IGF-1 signaling, contributing to vascular health, and its reduction post-menopause is associated with lower IGF-1 levels. In contrast, testosterone in men supports higher IGF-1 levels, but its direct effects on cerebrovascular protection are less clear. These sex-specific differences highlight the need to consider sex differences in research and therapeutic strategies related to IGF-1 and cerebrovascular health in aging populations.

Developing therapies that enhance IGF-1 signaling specifically within the neurovascular unit presents a significant opportunity for intervention. These targeted therapies aim to maximize vasoprotection while minimizing systemic side effects. Epigenetic modifications, such as DNA methylation, play a significant role in controlling IGF-1 gene expression. These modifications can be influenced by various lifestyle factors, including diet. Additionally, specific dietary compounds, such as polyphenols found in certain fruits and vegetables, may also modulate IGF-1 expression through epigenetic mechanisms. These findings highlight the dynamic nature of IGF-1 regulation and suggest that targeted dietary strategies could be used to optimize IGF-1 levels for cerebrovascular health. Long-term preclinical studies and clinical trials will be critical in assessing the effectiveness of IGF-1-based therapies in preventing CMH and other age-related cerebrovascular pathologies. These studies should focus on outcomes related to vascular health, cognitive function, and motor abilities, providing a comprehensive understanding of the potential benefits of IGF-1 therapies. Elevated circulating IGF-1 concentrations have been associated with an increased risk of certain types of cancers [74–76]. Understanding the interplay between IGF-1, IGFBPs, and dietary factors is essential for developing strategies that maximize the cerebrovascular benefits of IGF-1 while minimizing the associated cancer risks. While the current evidence is still in its early stages, further investigation into how exosomes [77, 78] might deliver bioactive IGF-1 to the cerebral microvasculature could open new avenues for therapeutic strategies aimed at preventing cerebrovascular diseases in aging populations.

As the elderly population suffers an increasing number of fall-related traumatic brain injuries (TBI) due to gait dysfunction, the incidence of CMHs is likely to rise. Even mild TBIs (mTBIs)—the most common type of TBI among the elderly—can induce CMHs [79, 80]. These injuries exacerbate the decline in IGF-1 levels [80–85] that naturally occurs with aging, creating a detrimental cycle where IGF-1 deficiency further impairs microvascular health. Reduced IGF-1 levels compromise the structural integrity of the cerebral vasculature, potentially making the microvessels more susceptible to rupture following repetitive mTBI. This situation highlights the TBI – IGF-1 deficiency – microvascular health – CMH axis, where mTBIs lead to a decline in IGF-1 levels [80–85], which in turn results in weakened vascular walls and an elevated risk of CMHs. Addressing this axis through targeted interventions that enhance IGF-1 signaling may provide a dual benefit of improving vascular resilience and reducing the incidence of CMHs post-TBI, thus protecting the elderly from the compounded risks of falls and vascular injury.

Investigating the potential of IGF-1 therapy in other cerebrovascular pathologies, such as ischemic stroke, CSVD, vascular cognitive impairment and dementia (VCID), and Alzheimer’s disease [86], could broaden the scope of its therapeutic applications. Biomarker development will play a pivotal role in advancing IGF-1 therapies. Identifying biomarkers that can predict the risk of CMH and monitor the effectiveness of IGF-1-based therapies will facilitate early intervention and treatment adjustments. Integrating personalized medicine approaches into IGF-1 therapy development offers another exciting avenue. Tailoring IGF-1-based therapies to individual patients based on their genetic makeup, IGF-1 levels, and specific cerebrovascular risk factors could enhance treatment efficacy and minimize side effects. By understanding and harnessing the vasoprotective role of IGF-1, we can better address the challenges posed by cerebrovascular aging. Advances in this field hold the promise of reducing the burden of cerebrovascular diseases, enhancing cognitive and motor functions, and ultimately contributing to healthier aging.

Overall, the future of IGF-1 research and therapy development is promising. Continued efforts to unravel the complexities of IGF-1 signaling and its impact on vascular health will be essential. By developing effective and targeted interventions, we can significantly improve the outcomes for aging populations at risk of cerebrovascular diseases, ensuring a better quality of life and increased longevity.
